# Dual mTOR/PI3K inhibition limits PI3K-dependent pathways activated upon mTOR inhibition in autosomal dominant polycystic kidney disease

**DOI:** 10.1038/s41598-018-22938-x

**Published:** 2018-04-03

**Authors:** Yang Liu, Martin Pejchinovski, Xueqi Wang, Xuebin Fu, Deborah Castelletti, Terry J. Watnick, Alexandre Arcaro, Justyna Siwy, William Mullen, Harald Mischak, Andreas L. Serra

**Affiliations:** 1 Institute of Physiology, University of Zürich, Zürich, Switzerland; 2 Mosaiques Diagnostics GmbH, Hanover, Germany; 3 Department of Nephrology, Second Military Medical University, Shanghai, China; 4 Department of Chemistry and Applied Biosciences, Molecular Pharmacology Unit, Swiss Federal Institute of Technology Zürich, Zürich, Switzerland; 5 Novartis Institute for Biomedical Research, Basel, Switzerland; 6 Division of Nephrology, University of Maryland School of Medicine, Baltimore, Maryland United States of America; 7 Department of Clinical Research, University of Bern, Bern, Switzerland; 8 Institute of Cardiovascular and Medical Sciences, University of Glasgow, Glasgow, UK; 9 Epidemiology, Biostatistics and Prevention Institute, University of Zürich, and Suisse ADPKD, Klinik Hirslanden Zürich, Switzerland; 10 Present Address: Department of Biomedicine, Cancer Immunology Laboratory, University Hospital Basel, Basel, Switzerland

## Abstract

Autosomal dominant polycystic kidney disease (ADPKD) is characterized by the development of kidney cysts leading to kidney failure in adulthood. Inhibition of mammalian target of rapamycin (mTOR) slows polycystic kidney disease (PKD) progression in animal models, but randomized controlled trials failed to prove efficacy of mTOR inhibitor treatment. Here, we demonstrate that treatment with mTOR inhibitors result in the removal of negative feedback loops and up-regulates pro-proliferative phosphatidylinositol 3-kinase (PI3K)-Akt and PI3K-extracellular signal-regulated kinase (ERK) signaling in rat and mouse PKD models. Dual mTOR/PI3K inhibition with NVP-BEZ235 abrogated these pro-proliferative signals and normalized kidney morphology and function by blocking proliferation and fibrosis. Our findings suggest that multi-target PI3K/mTOR inhibition may represent a potential treatment for ADPKD.

## Introduction

Autosomal dominant polycystic kidney disease (ADPKD) is the most common potentially lethal monogenetic hereditary kidney disease. It is characterized by progressive dilation of renal tubules that eventually form cysts, leading to kidney failure^1^. The loss of the major function of the kidneys due to cysts expansion has been largely associated with unexpected or asymptomatic well-know germ-line mutations, somatic mutations or even by reperfusion processes of ischemic tissue. These adverse effects are followed by glomerular hyperfiltration as a result of excess fluid accumulation^1,2^.

One of the key components is the mammalian target of rapamycin (mTOR) kinase which is a master regulator of protein synthesis and proliferation aberrantly activated during ADPKD onset^2,3^. Although treatment with mTOR inhibitors has shown positive results in preventing massive renal enlargement in a variety of polycystic kidney disease (PKD) animal models, clinical trials have not been able to show the same beneficial effect of mTOR inhibitors treatment in ADPKD patients^4–8^. It could be argued that a lack of good experimental study design, inappropriate drug dosage, inadequate therapy duration or patient stratification could be the reasons for such poor clinical outcomes. However, several studies investigated the dual negative feedback loop in a variety of human cancers: mTOR/S6K activation attenuates upstream phosphatidylinositol 3-kinase (PI3K) pathway activation, while treatment with mTOR inhibitors (rapamycin and its analogs) lead to a hyperactive insulin receptor substrate 1 (IRS-1)/PI3K pathway. This, in turn, increases the signaling toward the pro-proliferative extracellular signal-regulated kinases (ERK) and Akt pathways^9–12^. Based on these findings and our previous experimental work, we hypothesized that mTOR inhibition might also lead to compensatory up-regulation of the PI3K-dependent pathway in ADPKD by the release of mTOR controlled negative feedback loops that may attenuate the efficacy of mTOR inhibitors.

## Results and Discussion

**Figure 1:**
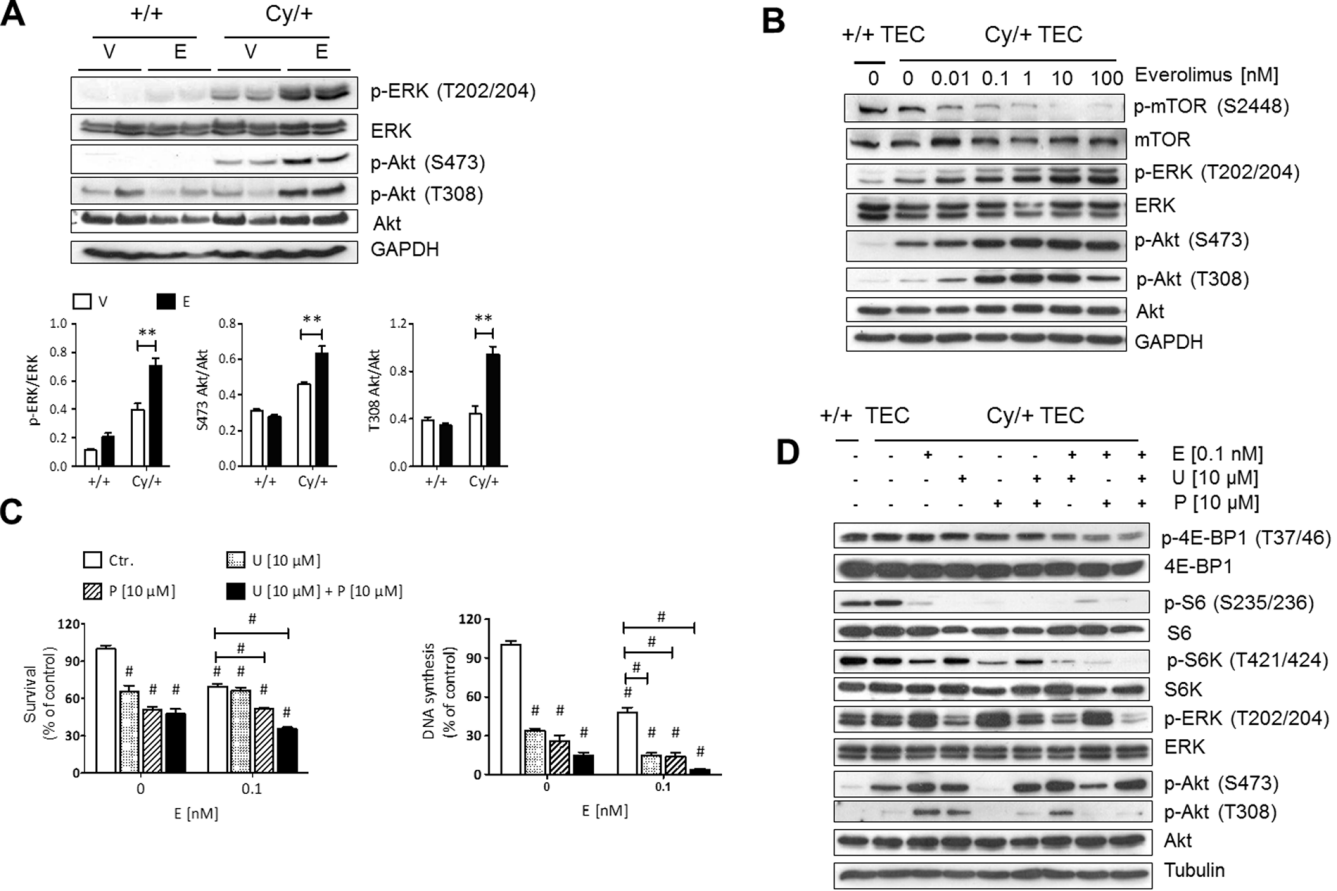
Mammalian TOR pathway blockage triggered dual PI3K-dependent feedback loops stemming from S6K to PI3K/ERK and to PI3K/Akt in PKD. (**A**) Han:SPRD rats whole kidney lysates western blot analysis and densitometry of PI3K/Akt and PI3K/ERK pathway readouts after 12 weeks everolimus (E) or vehicle (V) treatment (n = 4 per group). **p < 0.01 indicated by brackets. (**B**) mTOR upon treatment with indicated everolimus concentration for 48 h. (**C**) Cell viability (MTS, left panel) and DNA synthesis (BrdU, right panel) of Cy/ + renal primary tubular epithelial cells (TEC) after combined application of mTOR (everolimus, E), Akt (UO126, U) and ERK (perifosine, P) inhibitors for 48 h. ^#^p < 0.001 compared to control, except where indicated by brackets. (**D**) Western blot analysis of mTOR, PI3K/Akt and PI3K/ERK pathways after 48 h combination treatment with the indicated inhibitors in Cy/ + TEC. Graphs are representative of three independent experiments. These are cropped gels and the original gels are presented in Supplementary Fig. 6.

To explore our hypothesis we examined the effect of mTOR inhibitors on these dual negative feedback loops *in vitro* and in an animal model of PKD. For this purpose, we first treated Han:SPRD male rats, a well characterized strain (Cy/+) that resembles human ADPKD, with the rapamycin analog everolimus (gavage 3 mg/kg/day) from 4 to 16 weeks of age^8,13,14^. As a result, treatment with everolimus increased the activity of readouts of PI3K/Akt and PI3K/ERK in the polycystic kidney (Fig. 1A). Phosphorylation of T202/204-ERK, T308-Akt and S473-Akt were increased in polycystic kidneys of Cy/+ animals whereas in *wild type* animals these pathways were not activated by everolimus. In our next *in vitro analysis*, cultured Han:SPRD Cy/+ male renal tubular epithelial cells (TEC) were further investigated by everolimus treatment. This resulted in increased Akt and ERK activation and decreased activity of mTOR (phosphorylation of T389-S6K, T421/S424-S6K, S235/236-S6 and S240/244-S6, respectively) when used in a dose-dependent manner (Fig. 1B and D).

Previously although applying different methodology, Belibi and her colleagues carried out a similar study to determine the effect of rapamycin on PKD and the influence of the drug evaluation throughout alternation of pro-proliferative mammalian target of rapamycin complexes (mTORC1 and mTORC2) in female Han:SPRD cystic rats. The authors reported an increased S473-Akt in female Cy/+ after 9 weeks of ip injections of 0.2 mg/kg rapamycin per day. Interestingly, the opposite effect was observed when the same approach was applied in male Cy/+ rats, decreasing the S473-Akt activity suggesting a dose-dependent alteration of the S473-Akt regulation. Furthermore, differences in drug application method and treatment duration (gavage, 5 weeks treatment *versus* ip injection, 9 weeks treatment) may affect fibrosis and Akt expression levels^15–17^. Our *in vitro* and animal data highlighted the importance of mTOR inhibitors in assessing the effect on pro-proliferative signaling pathways in cystic pre-clinical animal models.

Currently, it is well-known that ADPKD is characterized by complex molecular interactions that contribute to cyst formations and further disease progression^18^. Often, due to the lack of appropriate translatability between humans and animal models, there are only a few pathological aspects that can be captured^19^. For this reason the initial PKD-associated signaling pathways were further investigated in ADPKD patients enrolled in the SUISSE ADPKD study^5^. While polycystic kidney specimens were not available from this trial, peripheral blood mononuclear cells (PBMCs) were isolated from patients before and after treatment with either sirolimus or standard care for 6 months^20–22^. Among sirolimus treated patients phosphorylation of ribosomal S6 protein was blocked whereas ERK phosphorylation was markedly increased and phospho-Akt was increased in 2 out of the total of 3 sirolimus treated ADPKD patients **(**Supplementary Fig. 1**)**. Analysis and interpretation of our laboratory data suggested similar effects of mTOR inhibition on pro-proliferative signaling in humans with ADPKD. Therefore, we further investigated the impact of mTOR signaling pathway upon treatment with mTOR inhibitors in Han:SPRD Cy/+ male renal tubular epithelial cells^21^. The set of everolimus, UO126, and perifosine inhibitors provoked triple inhibition of mTOR, ERK and Akt and in the same time considerably more effective loss of cell viability and inhibition of DNA synthesis than any double drug combination **(**Fig. 1C**)**. Western blot analysis confirmed the effect of each inhibitor on the respective pathways **(**Fig. 1D**)**.

**Figure 2:**
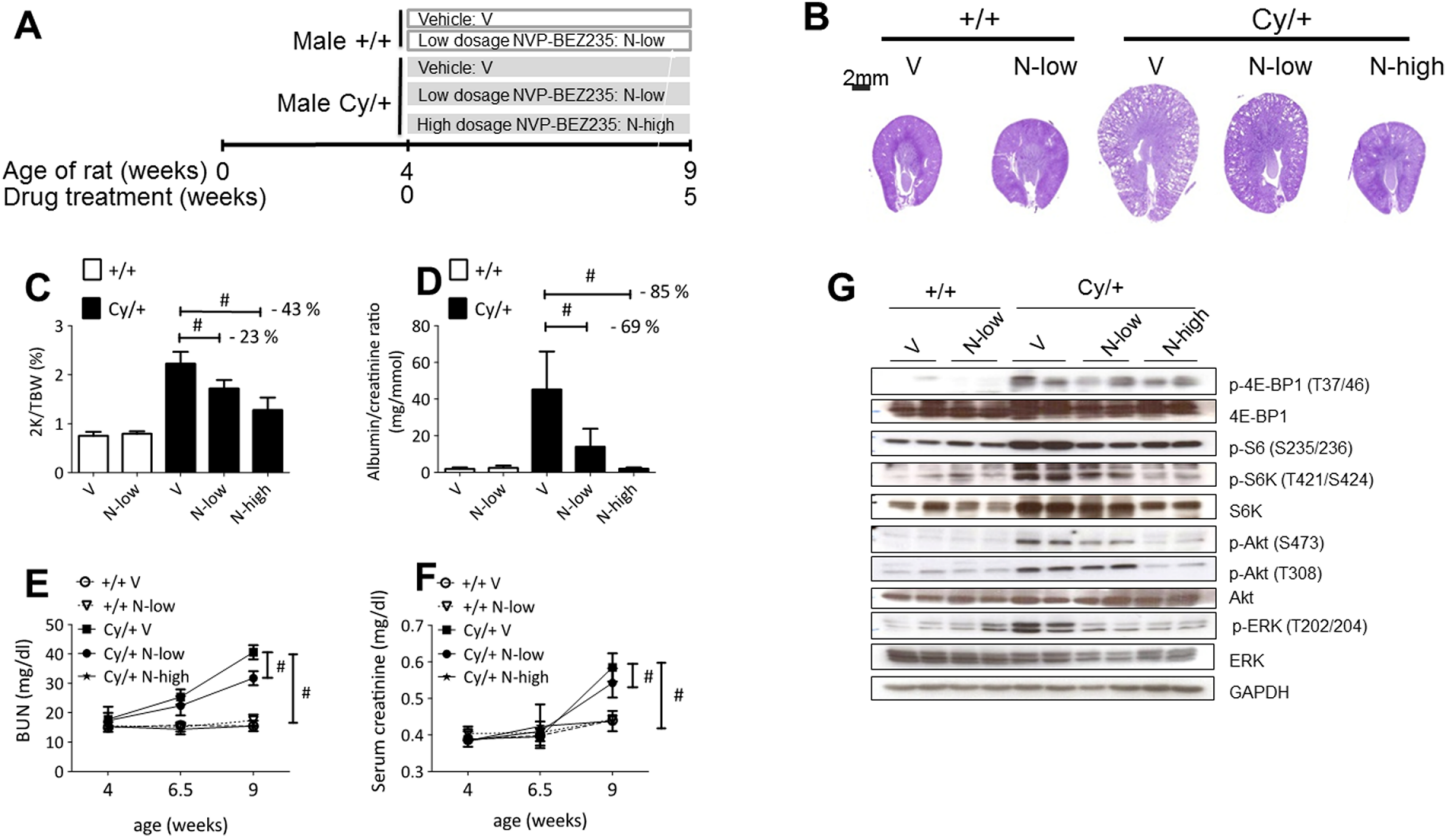
Efficacy of NVP-BEZ235 treatment on Han:SPRD rats renal function. (**A**) Study design scheme. Dose-dependent effect of NVP-BEZ235 on PKD progression. (**B**) Representative PAS stained kidney sections of NVP-BEZ235-treated and vehicle-treated Han:SPRD rats. Quantitative analysis of (**C**) two kidneys to total body weight ratio (2 K/TBW), (**D**) 24 h urine albumin to creatinine ratio, (**E**) BUN, (**F**) serum creatinine in Han:SPRD rats are displayed. **(G)** Western blot analysis of Han:SPRD whole kidney lysates for readouts of mTOR, PI3K/Akt and PI3K/ERK pathways after 5 weeks treatment with NVP-BEZ235-treated and vehicle-treated rat. Representative results of three independent experiments are shown. These are cropped gels and the original gels are presented in Supplementary Fig. 6. *p < 0.05, **p < 0.01, ^#^p < 0.001 indicated by brackets (mean ± SD, ANOVA). The number of rat per group is indicated in Supplementary Tables 1 and 2.

The suspension of abnormal cyst proliferation and expansion *in vivo* was investigated by administration of NVP-BEZ235 treatment, a dual mTOR/PI3K inhibitor with proven efficacy in various human cancer models^23–25^. In this analysis, we administrated low-dose (15 mg/kg/day, named N-low group) and high-dose (50 mg/kg/day, named N-high) NVP-BEZ235 to Han:SPRD male rats between 4 and 9 weeks of age **(**Fig. 2A**)**. Although this treatment reduced the body weight gain, in particular at the higher dose regimen, it had a dramatically positive effect on all aspects of the PKD disease burden: the kidney morphology (evaluated as the two kidneys weight/total body weight ratio, cyst volume, parenchymal fibrosis, and cell proliferation) was similar in NVP-BEZ235-treated PKD and in *wild type* animals **(**Fig. 2B,C Supplementary Fig. 4A–D and Supplementary Tables 1,2). In addition, renal function (blood urea nitrogen, serum creatinine concentration, urine albumin/creatinine ratio and plasma concertation) were all similar to that recorded in *wild type* animals (Fig. 2D–F and Supplementary Tables 1,2,7). We assessed t*he urinary proteomic/peptidomic molecular changes in the Han:SPRD rats to explore a potential biomarker associated with the beneficial effect of the NVP-BEZ2*3*5 treatment*^26^. (Supplementary Figure S6 and Supplementary Table 3). None of the animals died during the study and we observed a similar effect on body weight as reported for other mTORC1 inhibitors^8,27^. Notably, the renal function after 5 weeks of treatment was better in NVP-BEZ235-treated animals compared to everolimus-treated ones **(**Supplementary Table 4**)**^8^.

We elucidated the NVP-BEZ235 mode of action by analyzing the mTOR and its dual feedback loop pathway *in vivo*. NVP-BEZ235 blocked the mTOR pathway, as shown by the decrease of phospho-T421/S424-S6K and phospho-S235/236-S6. Expression of phospho-T308-Akt, phospho-S473-Akt and phospho-T202/204-ERK, used to monitor activation of the dual feedback loop, was prevented by the high-dose regimen, whereas the low-dose regimen was already sufficient to reduce the levels of phospho-S473-Akt and phospho-T202/204-ERK **(**Fig. 2G and Supplementary Fig. 3A**)**. Kidney tissue sections of untreated-Cy/ + rats showed that cysts-lining epithelial cells stained strongly positive for phospho-T202/204-ERK and phospho-S235/236-S6 in the cortex and in the corticomedullary region, whereas *wild type* and NVP-BEZ235-treated animals displayed only a mild and scattered positive staining **(**Supplementary Fig. 4A**)**. NVP-BEZ235 activates ERK in some cancer cells^28^ whereas we did not observe an ERK activation in our polycystic kidney models, nor in primary renal tubular epithelial cells **(**Fig. 2G and Supplementary Fig. 3B**)**^25,29,30^ Thus, dual mTOR/PI3K inhibition abrogates the up-regulation of Akt and ERK signaling pathways. Of note, NVP-BEZ235 had no effect on the pro-proliferative pathways, when analyzed in *wild type* treated kidneys, suggesting that the mode of action is specific for ADPKD **(**Fig. 2L**)**.

**Figure 3:**
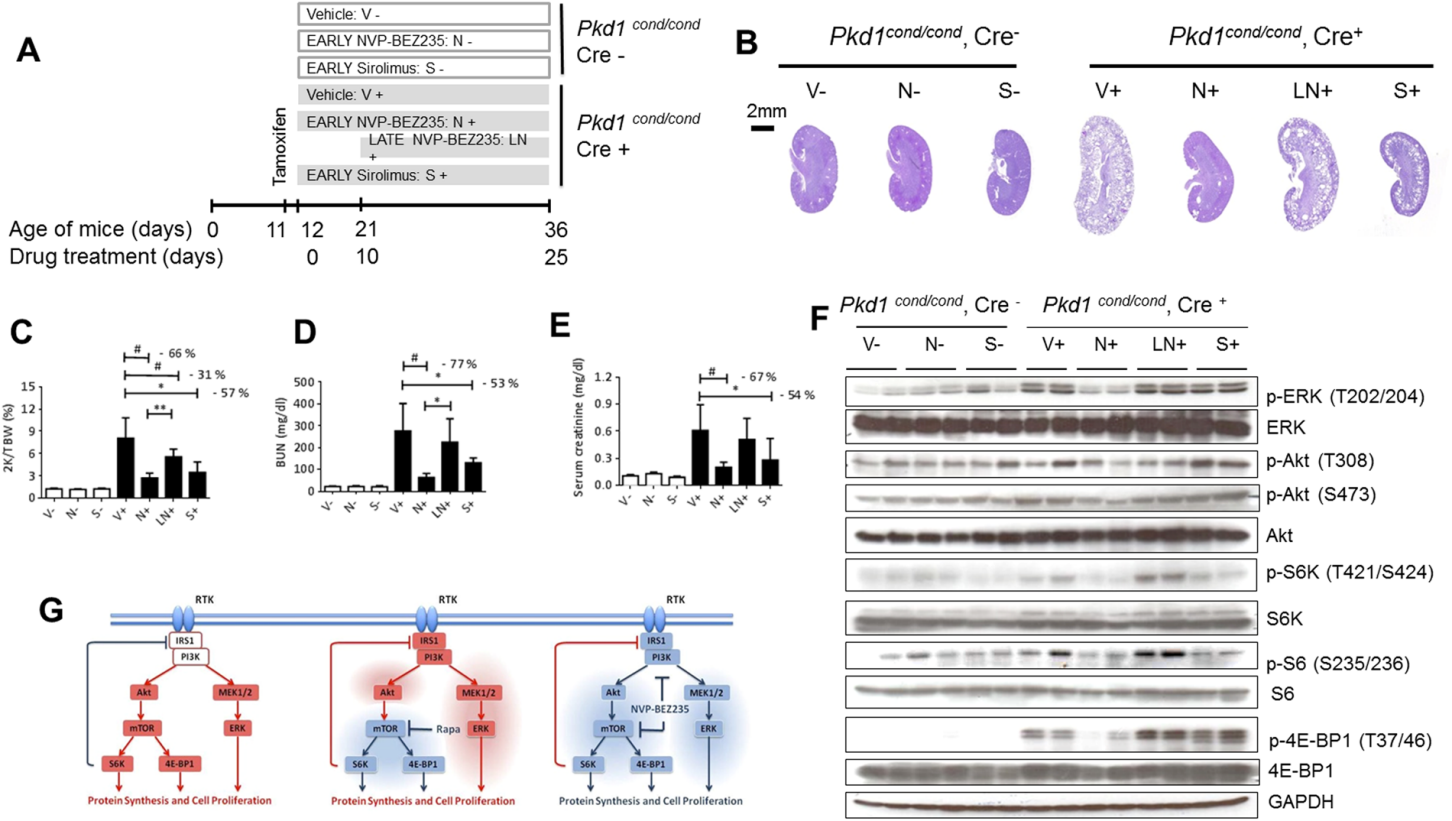
Efficacy of NVP-BEZ235 and sirolimus treatment in *Pkd1* conditional knockout mice. (**A**) Study design scheme. (**B**) Representative PAS stained kidney sections of NVP-BEZ235-treated, sirolimus-treated and vehicle-treated mice. Quantitative analysis of (**C**) two kidneys to total body weight ratio (2 K/TBW), (**D**) BUN, (**E**) serum creatinine, *p < 0.05, **p < 0.01, ^#^p < 0.001 indicated by brackets (mean ± SD, ANOVA). The number of mice per group is indicated in Supplementary Tables 4 and 5. (**F**) Whole kidney lysates Western blot analysis of mTOR, PI3K/Akt and PI3K/ERK pathways (**G**) Schematic representation of the pathways examined in the study. mTOR pathway is abnormally activated in polycystic kidneys (left panel). Treatment with mTOR inhibitor results in a hyperactive PI3K-dependent pathway, increasing the signal toward the PI3K/Akt and PI3K/ERK pathway (middle panel). Dual mTOR/PI3K inhibitor NVP-BEZ235 may provide a therapeutic benefit for ADPKD by abrogating the activation of PI3K/Akt and PI3K/ERK pathways and in turn block the mTOR pathway (right panel). Red denotes activated; blue, inhibited. These are cropped gels and original gels are presented in the Supplementary Fig. 6.

To further assess the relevance of dual mTOR and PI3K-dependent pathway inhibition as a therapy for PKD, we compared the efficacy of NVP-BEZ235 and sirolimus in an orthologous ADPKD mouse model with a conditionally inactivated *Pkd1* gene^31^. Firstly, we induced cystogenesis by tamoxifen intraperitoneal (ip) injection into pups at postnatal day 11, then deterioration of the kidney function occurred over 4 to 6 weeks^32^. We assessed the efficacy of NVP-BEZ235 for two different treatment initiation time points: At an early disease state with 6 mg/kg dosage before weaning between day 12 and day 35 and at the same time points with 9 mg/kg after weaning (N+ group). Over the same time period, sirolimus with 3 mg/kg dosage (S+ group) was also applied. The late disease stage drug administration was evaluated between day 21 and 35 with the ip method of injection and dosage of 9 mg/kg NVP-BEZ235 (LN+ group) (Fig. 3A). Our preclinical results showed a marked beneficial effect at early disease stage mirrored by reducing kidney volume and preserving further cysts enlargement **(**Fig. 3B,C). Furthermore early treatment preserved body weight (Supplementary Fig. 3C); prevented kidney function decline (blood urea nitrogen levels (Fig. 3D) and serum creatinine levels (Fig. E)): inhibited cytogenesis (Supplementary Fig. 5B), fibrogenesis (Supplementary Fig. 5C) and cell proliferation (Supplementary Fig. 5D). Sirolimus (S+ group) was less efficient than NVP-BEZ235 (N+ group) with respect to cystogenesis, renal function and proliferation (Fig. 3B–E and Supplementary Fig. 5B–D**)**. Western blot analysis displayed reduced expression of phospho-S6K, phospho-S6 and phospho-4EBP and no up-regulation phospho-ERK and phospho-Akt during early stage PKD (N+ group) whereas sirolimus (S+ group) triggered Akt activation (Fig. 3F and Supplementary Fig. 3). Late NVP-BEZ235-treated mice (LN+ group) exhibited renal function decline among all three treatment groups (Fig. 3B–E), and Western blot analysis indicated increased gene expression of phospho-T202/204-ERK, phospho-T421/S424-S6K, and phospho-S235/236-S6 (Supplementary Fig. 5A and Supplementary Tables 5,6).

Ren and his colleagues investigated safety and efficacy of NVP-BEZ235 in PCK rats, an established autosomal recessive polycystic kidney (ARPKD) model. The NVP-BEZ235 had no beneficial effect on cystogenesis in the ARPKD rodent model^33^. In another study, Pollizi and the colleagues have shown that NVP-BEZ235 and everolimus had equivalent effects in suppressing the development of cystadenomas in a TSC2 mouse model^34^. In clinical practice, assessment of safety and efficiency of NVP-BEZ235 in 20 patients with locally advance or metastatic translation cell carcinoma (TCC) after failure of platinum-based therapy showed only moderate beneficial effect and severe drug-related complications in the majority of the patients^35^. We assume that even greater toxicity would be observed in humans required for ADPKD given the longer term dosing. Therefore, careful risk stratification is of outmost importance to identify patients eligible for treatments to balance therapeutic efficacy and possible adverse effects.

Our signaling pathway analysis after NVP-BEZ235 treatment suggests a feedback loop. Indeed, a recent study has demonstrated that there is a new feedback loop compensatory activation of Rictor and S473-Akt via FoxO-mediated upregulation after NVP-BEZ235 treatment which can be used as a novel therapeutic target in human renal carcinoma cells^36^. Another study has shown that NVP-BEZ235 is a potential inhibitor of ataxia telangiectasia mutated (ATM) and DNA-dependent protein kinase (DNA-PK), which are unexplored but may implicate the DNA double strand break (DNA-DSB) repair pathways in PKD^37–39^.

Our findings that sirolimus treatment increased renal S473-Akt level in the orthologous ADPKD mouse model was not observed in the study of Shillingford and colleagues. The difference could be associated with the PKD1^cond/cond^:Nestin^Cre^ mice used by Shillingford. In this mouse model renal cysts arise predominantly from the collecting duct and distal tubules whereas in our PKD1^cond/cond^:Tamoxifen-Cre+ mice cysts develop in all nephron segments and renal dysfunction occurs over 4 to 6 weeks. Future experimental studies using a slow progressive disease model and PKD1^cond/cond^:Nestin^Cre^ model is needed to further elucidate the effect of NVP-BEZ235 on the signaling pathway associated with cystogenesis and cyst progression^6,32^.

Our study has to be interpreted in the context of the study design. Firstly, the experiments in the animal models are performed in relatively small group sizes. Secondly, only males were used for the Han:SPRD rats due to the different rate of disease progression in male and female rats. Thirdly, the effect of NVP-BEZ235 at high doses on the body weight of Han:SPRD rat suggests toxicity and thus warrants careful consideration before applying this drug in clinical trials. Our pre-clinical data indicated the beneficial effect of the dual mTOR/PI3K inhibitor NVP-BEZ235 and highlighted the need for well-designed clinical trials to explore NVP-BEZ235 as a potential treatment in ADPKD patients.

## Conclusion

In conclusion, blockage of the mTOR pathway, by using mTOR inhibitors, triggers up-regulation of pro-proliferative PI3K-dependent pathways, either through the PI3K/Akt or PI3K/ERK pathway in PKD. Dual mTOR/PI3K inhibition specifically prevents the activation of these feedback loops and (Fig. 3G), when applied in early stage disease, this treatment normalizes the renal function and the morphology of ADPKD non-orthologous rat and orthologous mice models.

## Methods

The protocols and methods used in this study were in accordance with the EU Directive 2010/63/EU-On the protection of animal for experimental and other scientific purposes. All standards, international convention and declaration were supervised by the European Group on Ethics and Protection of Animals.

### Experimental animals and study design

#### Han:SPRD rat

The Han:SPRD rat colony was established in our animal facility from a litter, which was obtained from the Rat Resource and Research Center (Columbia, MO). Heterozygous cystic (Cy/+) and wild-type (+/+) male rats were used in this study. Figure 2A display the rat study set up: we applied by gavage NVP-BEZ235 (Novartis) at low-dose (15 mg/kg/day), high-dose (50 mg/kg/day) and vehicle solution (90% PEG300 and 10% 1-mehtyl-2-pyrrolidinone, Sigma-Aldrich) from 4 to 9 weeks of age. Blood was collected at 4, 6.5 and 9 weeks of age for determination of serum creatinine and BUN levels. 24 h urine samples were collected in metabolic cages one day before the rats were killed. We applied by gavage everolimus (E, 3 mg/kg/day) and vehicle solution (V) from 4 to 16 weeks of age. Rats were anesthetized with isoflurane and kidneys were harvested for further analysis.

#### Pkd1 conditional knockout mouse

Professor G.G Germino supplied the Pkd1 conditional knockout mouse. We

induced Cre recombinase activity by intraperitoneal injection of tamoxifen (1.25 mg/10 g, dissolved in corn oil (Sigma-Aldrich) at day 11 of age into pups. Figure 3A display the mice study set up: The mice were randomly allocated to seven groups; *Pkd1cond/cond*, Cre + vehicle (V+), *Pkd1cond/cond*, Cre+ early-phase NVP-BEZ235 (N+), *Pkd1cond/cond*, Cre+ late-phase NVP-BEZ235 (LN+), *Pkd1cond/cond*, Cre+ early-phase sirolimus (S+), *Pkd1cond/cond*, Cre- vehicle (V−), *Pkd1cond/cond*, Cre- early-phase NVPBEZ235(N−), and *Pkd1cond/cond*, Cre- early-phase sirolimus (S−). For the N+ and Ngroups, animals were treated with 6 mg/kg/day NVP-BEZ23 from day 12 to day 20, and later with 9 mg/kg/day until day 35. For the S+ and S− groups, 3 mg/kg/day sirolimus was administrated from day 12 to day 35. For the LN+ group, mice were

treated with 9 mg/kg/day NVP-BEZ235 from day 21 to day 35. NVP-BEZ235, sirolimus and vehicle solutions were administrated by intraperitoneal injections. 24 h urine samples were collected by metabolic cage at day 35. Mice were anesthetized with isoflurane at day 36, and blood was obtained by cardiac puncture for determination of serum creatinine and BUN levels and kidneys were harvested for further analysis.

### Biochemical analysis

Rat and mice serum creatinine and BUN levels were detected with UniCel® DxC 800 Synchron® Clinical (Zurich Integrative Rodent Physiology). The GenWay Albumin Elisa Kits for rat were used for rat urine albumin concentration measurement, according to the manufacturer’s protocol.

### Proteomic analysis

PKD rat urine aliquots were thawed immediately before use and 150 μl were mixed with 150 μl solution containing 2 M urea, 10 mM NH4OH and 0,02% SDS. Subsequently, aliquots were filtered with Centrisart ultracentrifugation filters (20 kDa MWCO, Sartorius, Goettingen, Germany) to remove the higher molecular weight proteins. Obtained filtrate was desalted using a NAP 5 gel filtration column (GE Healthcare Bio Science, Uppsala, Sweden) to remove urea and electrolytes. Samples were lyophilized and stored on 4 0 C. Shortly before CE-MS analysis, the aliquots were re-suspended in 10 μl HPLC grade H2O. CE-MS technology was performed using P/ACE MDQ capillary electrophoresis system (Beckman Coulter, Fullerton, CA), on-line couple to a Micro-TOF MS (Bruker Daltonic, Bremen, Germany). Spectra were accumulated every 3 s over m/z range of 350–3000 Da. Details of the CE-MS technology has been described previously^40^. Proteomic data was further processed by MosaVisu software package in order to deconvolve mass spectral ion peaks representing identical molecules at different charge states into single masses. Normalization of migration time and ion signal intensity (amplitude) was achieved by using internal endogenous peptide standards for biomarker quantification. Based on this approach using “housekeeping” peptides, calculation of the relative abundance allows both correction of analytical variance and correction for different dilution levels of individual urine samples. All detected polypeptides, characterized by their molecular mass (kDa), CE-migration time (min) and ion signal intensity, were matched and annotated by usage of Microsoft SQL database. Peptide marker panel was established using support vector machine (SVM)-based MosaCluster software package^41^. The reported p-values of the identified potential peptide markers were calculated with Wilcoxon Rank Sum test followed by adjustment of multiple testing with the method described by Benjamini and Hochberg^42^. Peptides detected with frequency of ≥90% in at least one of the animal groups were considered for statistical analysis.

To obtain sequence informations, urine samples were analyzed on a Dionex Ultimate 3000 RSLS nano flow system (Dionex, Camberly UK) couple to an Orbitrap Velos FTMS (Thermo Fisher, Bremen, Germany). The fragmentation method was HCD at 35% collision energy. FTMS analyzer was selected with 60 000 resolution for MS1 and 7500 resolution for MS2. Peptides and proteins were searched against UniProt rat database and identified with SEQUEST spectral algorithm (Thermo), without any enzyme specificity. Precursor mass tolerance and fragment mass tolerance were 10 ppm and 0.05 Da, respectively. No fixed modification was selected and oxidation of methionine and proline were set up as variable modifications. High confidence peptides with Xcorr ≥1.9 and rank 1 were accepted as most valid for identification of the peptide markers^43^. Mass deviation was set below ±80 ppm.

### Statistics

Data are presented as mean ± SD. Statistical differences between treatment groups were performed by the unpaired two-tailed *t*-test using GraphPad Prism version 4.0 (GraphPad). P < 0.05 was considered to be statistically significant.

### Study approval

The study was approved and ruled under animal license number 22/2010 obtained from Zürich Veterinary Office.

### Availability of data and materials

The raw datasets used and/or analyzed during the current study are available from the corresponding author on reasonable request.

## Electronic supplementary material

Supplementary Information
