# Supplementary material for: Dual mTOR/PI3K inhibition limits PI3K-dependent pathways activated upon mTOR inhibition in autosomal dominant polycystic kidney disease
Source: Sci Rep. 2018 Apr 3;8:5584. doi: 10.1038/s41598-018-22938-x (PMC5882886; doi:10.1038/s41598-018-22938-x)
Supplement: Supplementary file 1 — Supplementary Information [file 41598_2018_22938_MOESM1_ESM.pdf]

Dual mTOR/PI3K inhibition limits PI3K-dependent pathways activated upon mTOR inhibition in autosomal dominant polycystic kidney disease

Yang Liu[1¶*], Martin Pejchinovski[2*], Xueqi Wang[3], Xuebin Fu[4], Deborah Castelletti[5], Terry J. Watnick[6], Alexandre Arcaro[7], Justyna Siwy[2], William Mullen[8], Harald Mischak[2,8], Andreas L. Serra[9]

1. Institute of Physiology, University of Zurich, Switzerland
2. Mosaiques Diagnostics GmbH, Hanover, Germany
3. Department of Nephrology, Second Military Medical University, Shanghai, China
4. Department of Chemistry and Applied Biosciences, Molecular Pharmacology Unit, Swiss Federal Institute of Technology Zurich, Switzerland
5. Novartis Institute for Biomedical Research, Basel, Switzerland
6. Division of Nephrology, University of Maryland School of Medicine, Baltimore, Maryland, United States of America
7. Department of Clinical Research, University of Bern, Switzerland
8. Institute of Cardiovascular and Medical Sciences, University of Glasgow, Glasgow, UK
9. Epidemiology, Biostatistics and Prevention Institute, University of Zürich, and Suisse ADPKD, Klinik Hirslanden Zürich, Switzerland

¶ Present address: **Department of Biomedicine, Cancer Immunology Laboratory, University Hospital Basel, Basel, Switzerland**
* These authors contributed equally to this work

Correspondence to:
Andreas L. Serra, MD, MPH
Epidemiology, Biostatistics and Prevention Institute
University of Zürich
Hirschengraben 84
CH-8001 Zurich
Tel. +41 44 634 46 11
Email: andreas.serra@uzh.ch

SUPPLEMENTARY EXPERIMENTS

Urine rat proteome/peptidome analysis

To test whether the urinary proteomic signature is different in rat PKD animal model, we aimed to assess the urine proteome in wild type placebo and cystic placebo groups and evaluate the ability of the urinary biomarkers to detected dose-dependent drug response in PKD rats. For this purpose, urinary proteome analysis using capillary electrophoresis coupled to mass spectrometry (CE-MS) was performed. This enabled the selection of 30 peptides with significantly different abundance in wild type and cystic placebo animals. Combination of the 30 peptides into a predictive peptide classifier using machine learning algorithm clearly separated control and placebo treated cystic animals **(Supplementary Fig.7A, left plot).** When applied to higher dose and lower dose NVP-BEZ235 treated PKD animals, the score of this peptide marker panel reached the value of control animals suggesting normalization of the disease-induced signature (**Supplementary Fig.7A, right plot**). Using tandem mass-spectrometry, we were able to obtain sequences for 13 out of these 30 disease-associated peptide markers **(Supplementary Table 3).** Majority of the identified rat peptide markers were fragments of various collagens indicating reorganization of the extracellular matrix (ECM) system, triggered during cyst formation and expansion as a result of the NVP-BEZ235 treatment. The beneficial effect of the NVP-BEZ235 treatment on the rat urinary proteome is also graphically displayed as complied contour plots by the amplitudes of these 30 individual peptides (**Supplementary Fig.2B**).

Figure S1

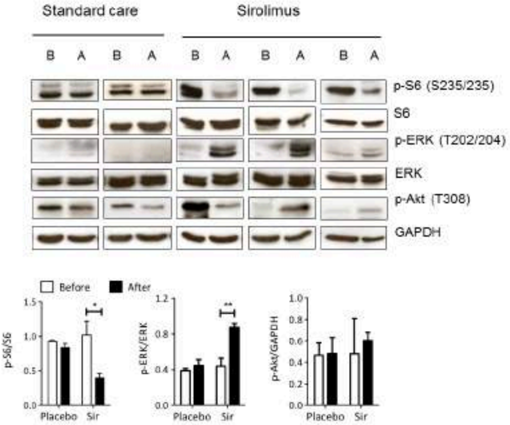

Standard care
Sirolimus
B A B A B A B A B A
p-S6 (S235/235)
S6
p-ERK (T202/204)
ERK
p-Akt (T308)
GAPDH
Before
After
p-S6/S6
p-ERK/ERK
p-Akt/GAPDH
Placebo
Sir

Figure S2

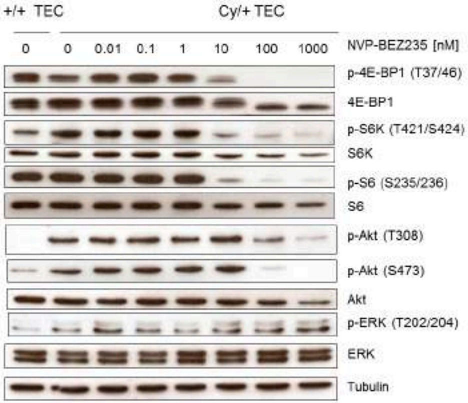

+/+ TEC
Cy/+ TEC
0
0
0.01
0.1
1
10
100
1000
NVP-BEZ235 [nM]
p-4E-BP1 (T37/46)
4E-BP1
p-S6K (T421/S424)
S6K
p-S6 (S235/236)
S6
p-Akt (T308)
p-Akt (S473)
Akt
p-ERK (T202/204)
ERK
Tubulin

Figure S3

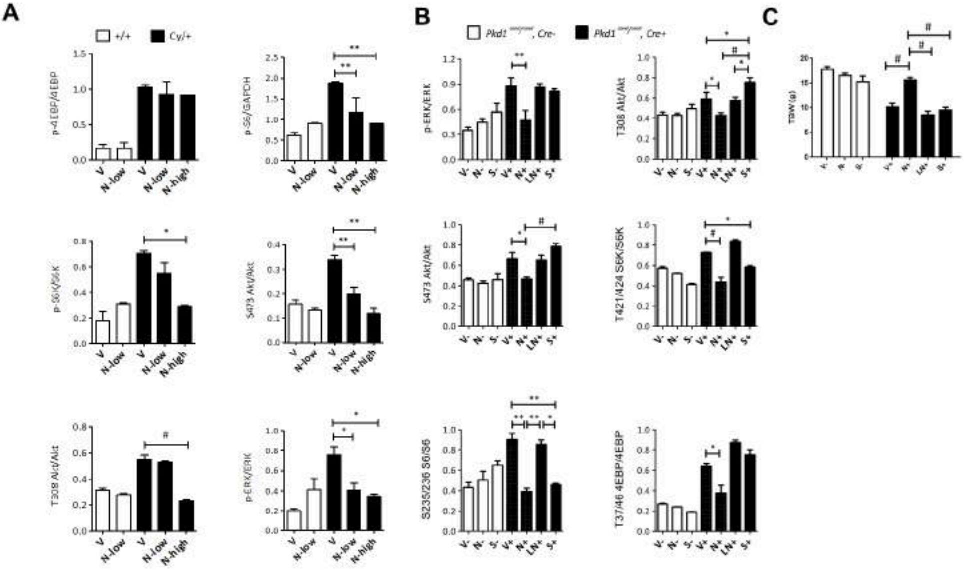
A
+/+
Cy/+
p-4EBP/4EBP
p-S6/GAPDH
p-S6K/S6K
S473 Akt/Akt
T308 Akt/Akt
p-ERK/ERK
V
N-low
N-high
B
Pkd1, Cre-
Pkd1, Cre+
p-ERK/ERK
T308 Akt/Akt
S473 Akt/Akt
T421/424 S6K/S6K
S235/236 S6/S6
T37/46 4EBP/4EBP
V-
N-
S-
V+
N+
LN+
S+
C
TBW (g)

Figure S4

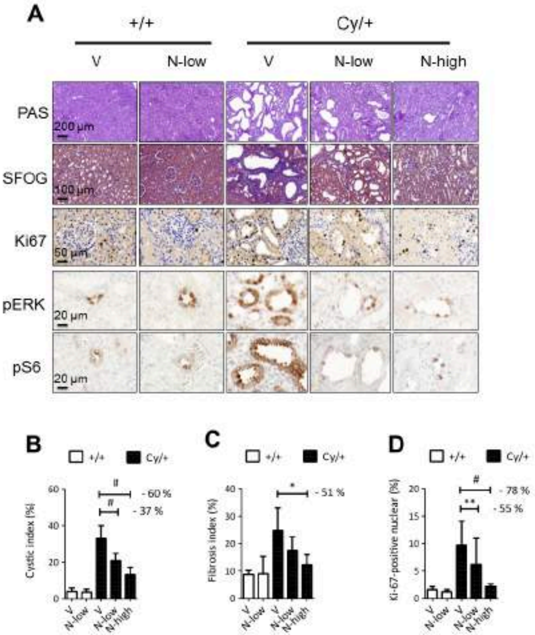

A
+/+
Cy/+
V
N-low
V
N-low
N-high
PAS
200 µm
SFOG
100 µm
Ki67
50 µm
pERK
20 µm
pS6
20 µm
B
+/+
Cy/+
Cystic index (%)
- 60 %
- 37 %
C
Fibrosis index (%)
- 51 %
D
Ki-67-positive nuclear (%)
- 78 %
- 55 %

Figure S5

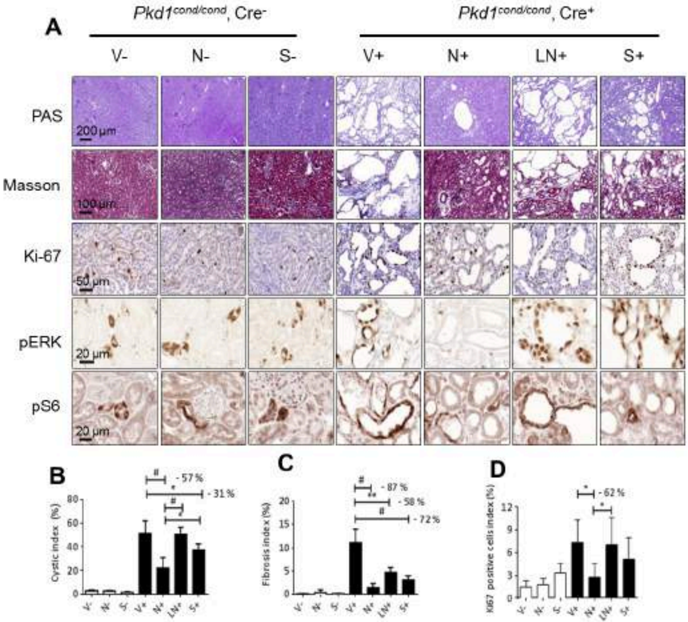
A
Pkd1cond/cond, Cre-
Pkd1cond/cond, Cre+
V-
N-
S-
V+
N+
LN+
S+
PAS
Masson
Ki-67
pERK
pS6
200 µm
100 µm
50 µm
20 µm
20 µm
B
Cystic index (%)
- 57 %
- 31 %
C
Fibrosis index (%)
- 87 %
- 58 %
- 72 %
D
Ki67 positive cells index (%)
- 62 %

Figure S6

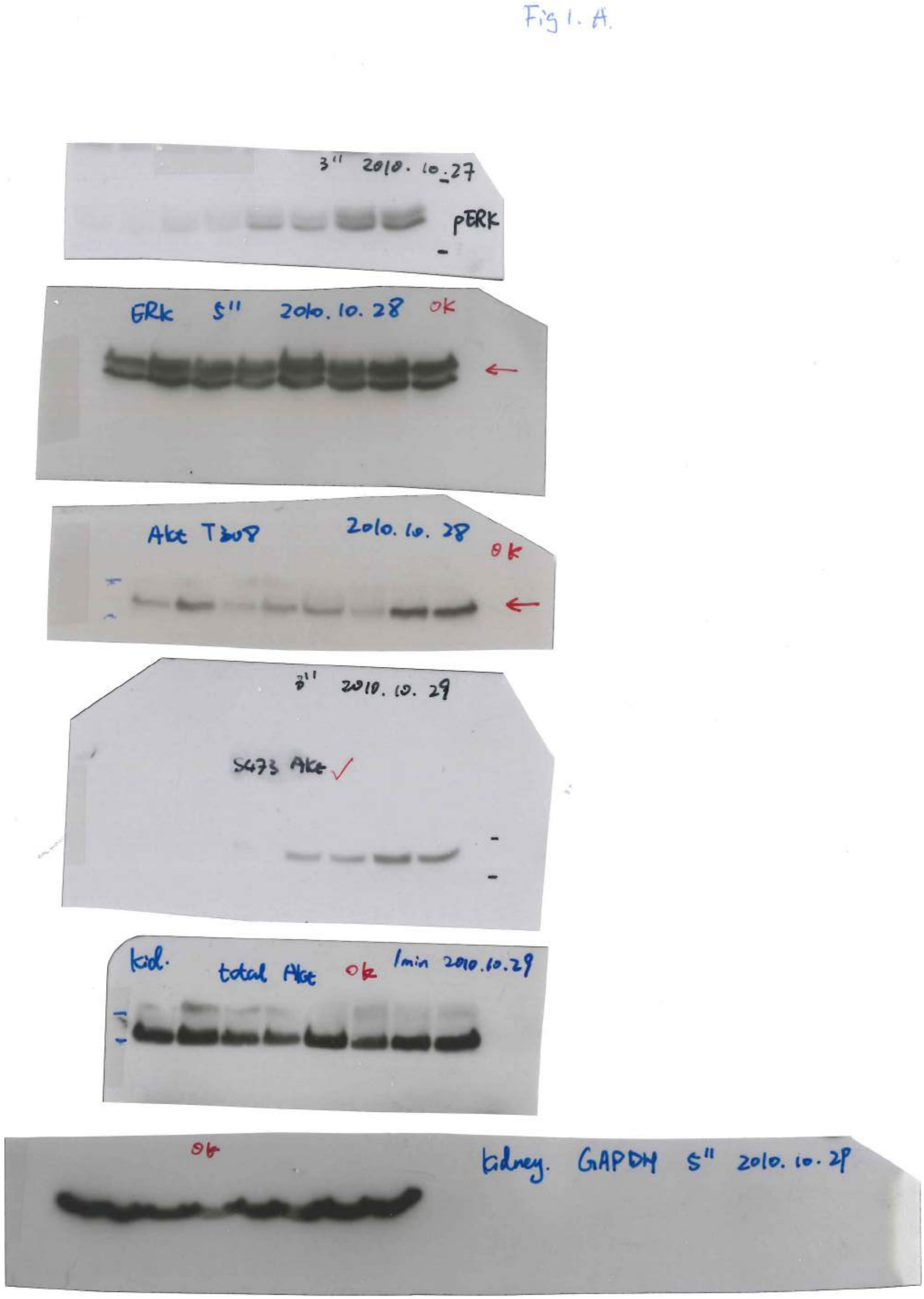
Fig 1. A.
3'' 2010. 10. 27
pERK
ERK 5'' 2010. 10. 28 ok
Akt T308 2010. 10. 28 ok
3'' 2010. 10. 29
S473 Akt
kid. total Akt ok 1min 2010.10.29
ok
kidney. GAPDH 5'' 2010. 10. 28

Fig. 1 B

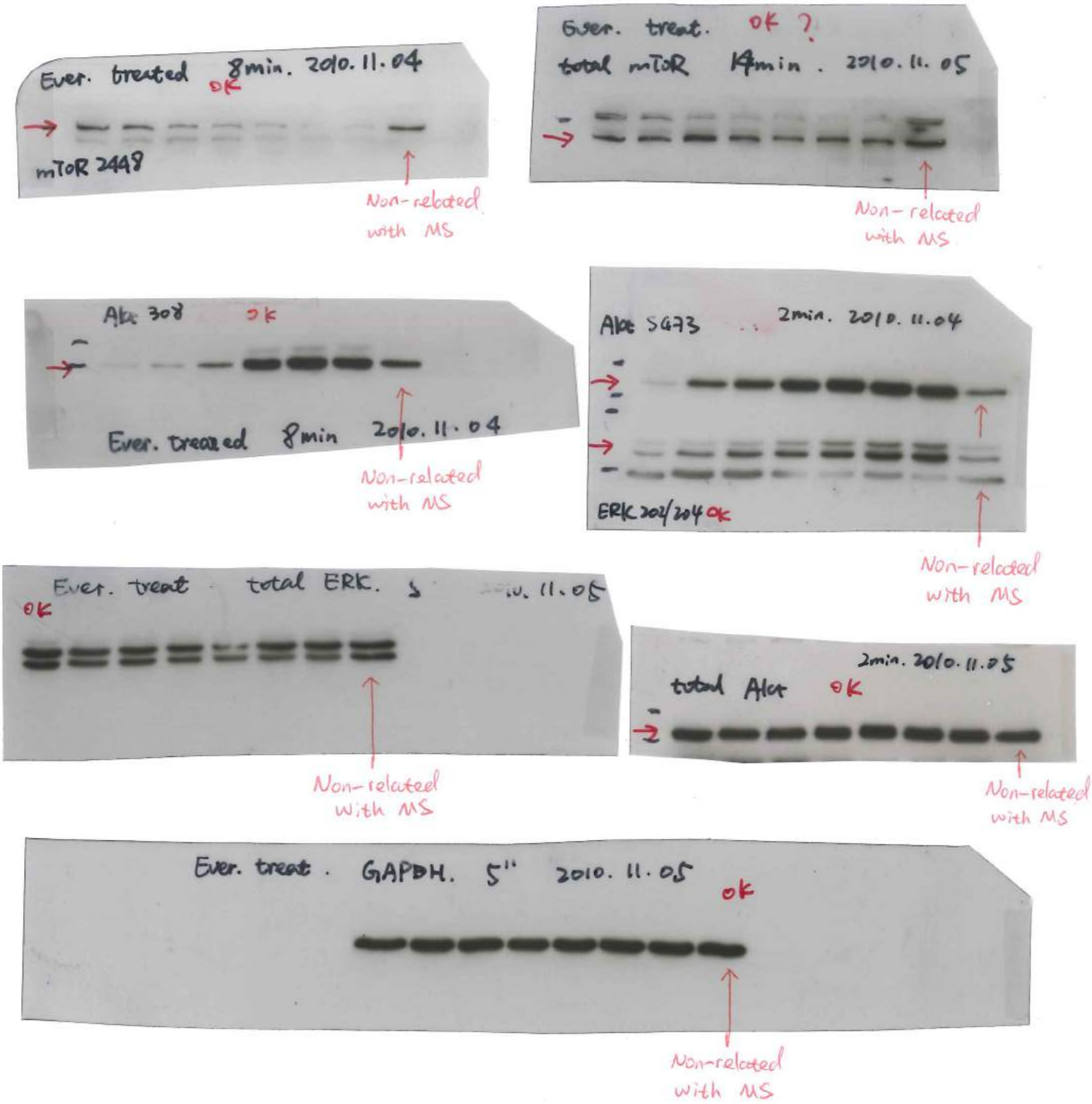

Ever. treated 8min. 2010. 11. 04
OK
mTOR 2448
Non-related with MS
Ever. treat. OK ?
total mTOR 14min. 2010. 11. 05
Non-related with MS
Akt 308 OK
Ever. treated 8min 2010. 11. 04
Non-related with MS
Akt S473 2min. 2010. 11. 04
ERK 202/204 OK
Non-related with MS
Ever. treat total ERK.
OK
Non-related with MS
2min. 2010. 11. 05
total Akt OK
Non-related with MS
Ever. treat. GAPDH. 5'' 2010. 11. 05
OK
Non-related with MS

For Figure 1. D

non-relative with manuscript.

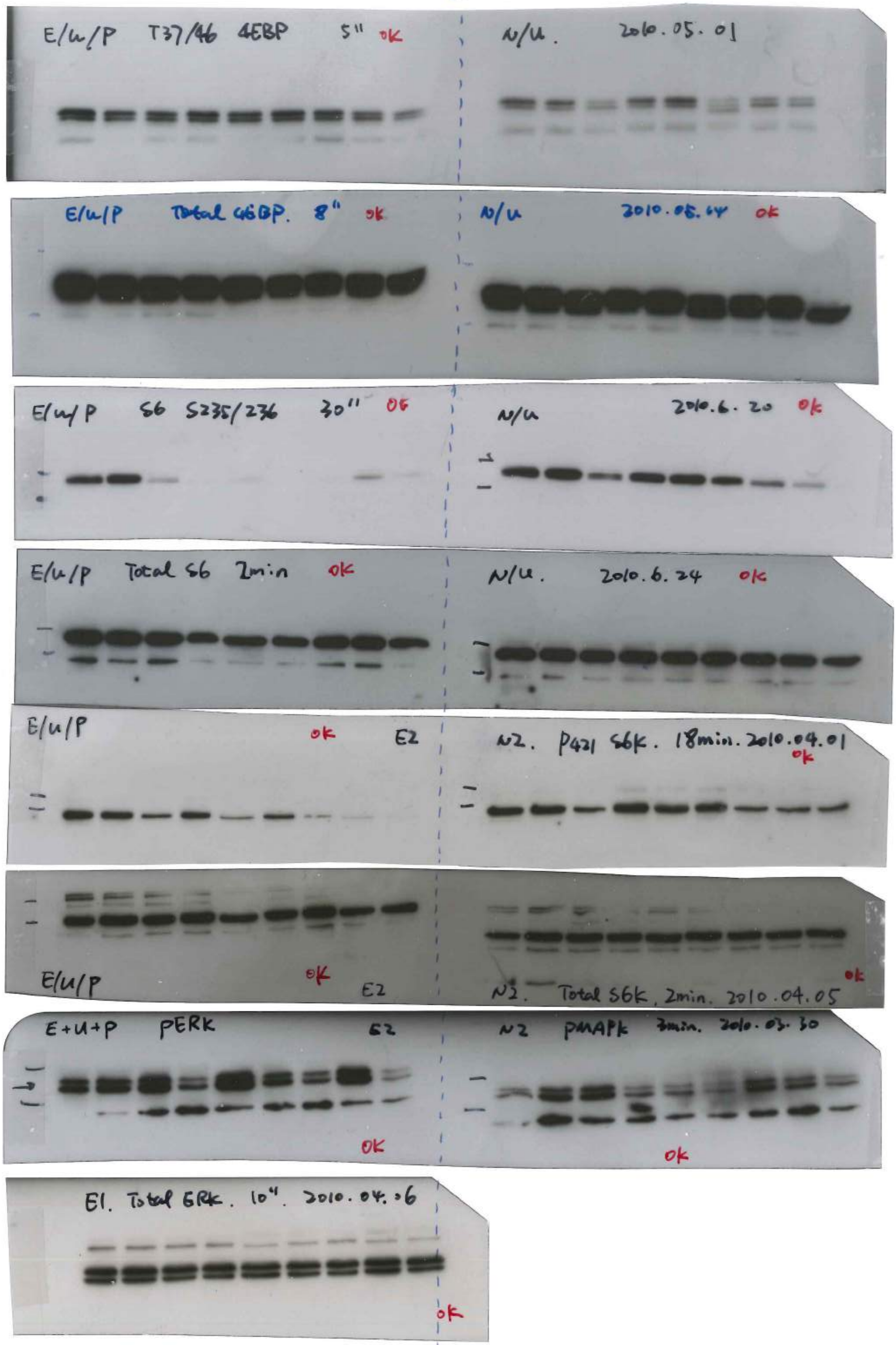

Figure 1. D

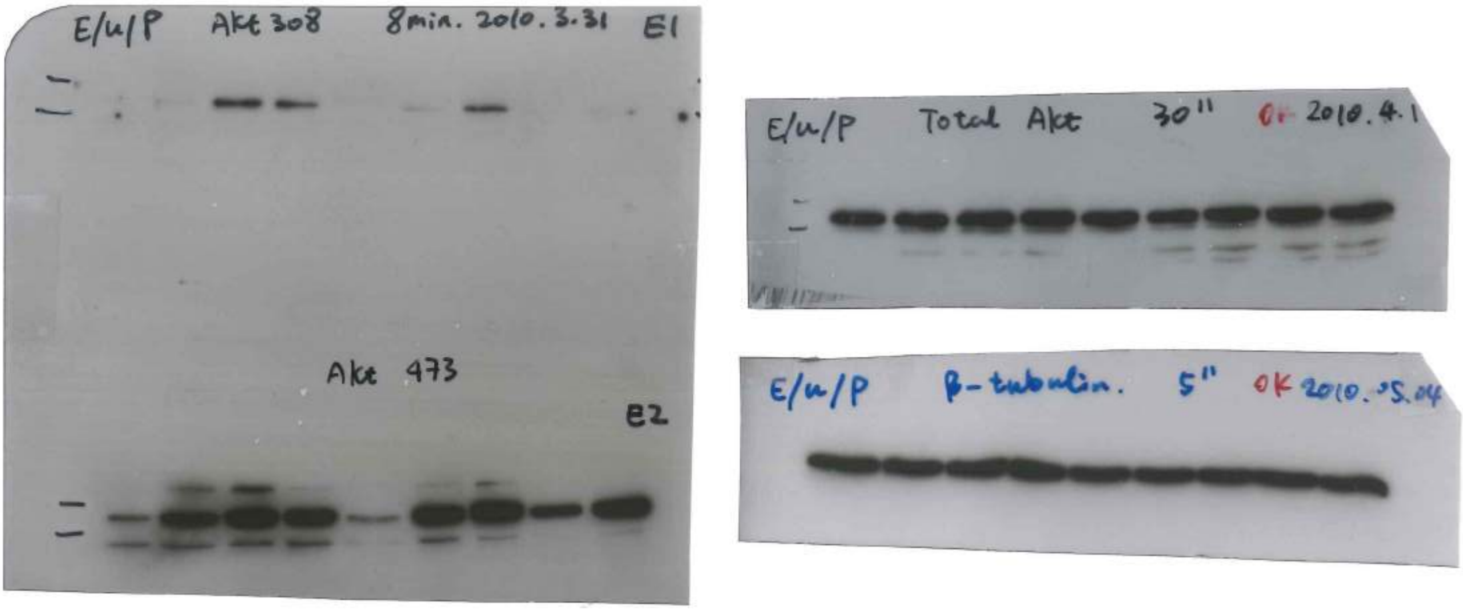
E/u/P Akt 308 8min. 2010.3.31 E1
Akt 473
E2
E/u/P Total Akt 30'' ok 2010.4.1
E/u/P β-tubulin. 5'' OK 2010.05.04

figure 2L

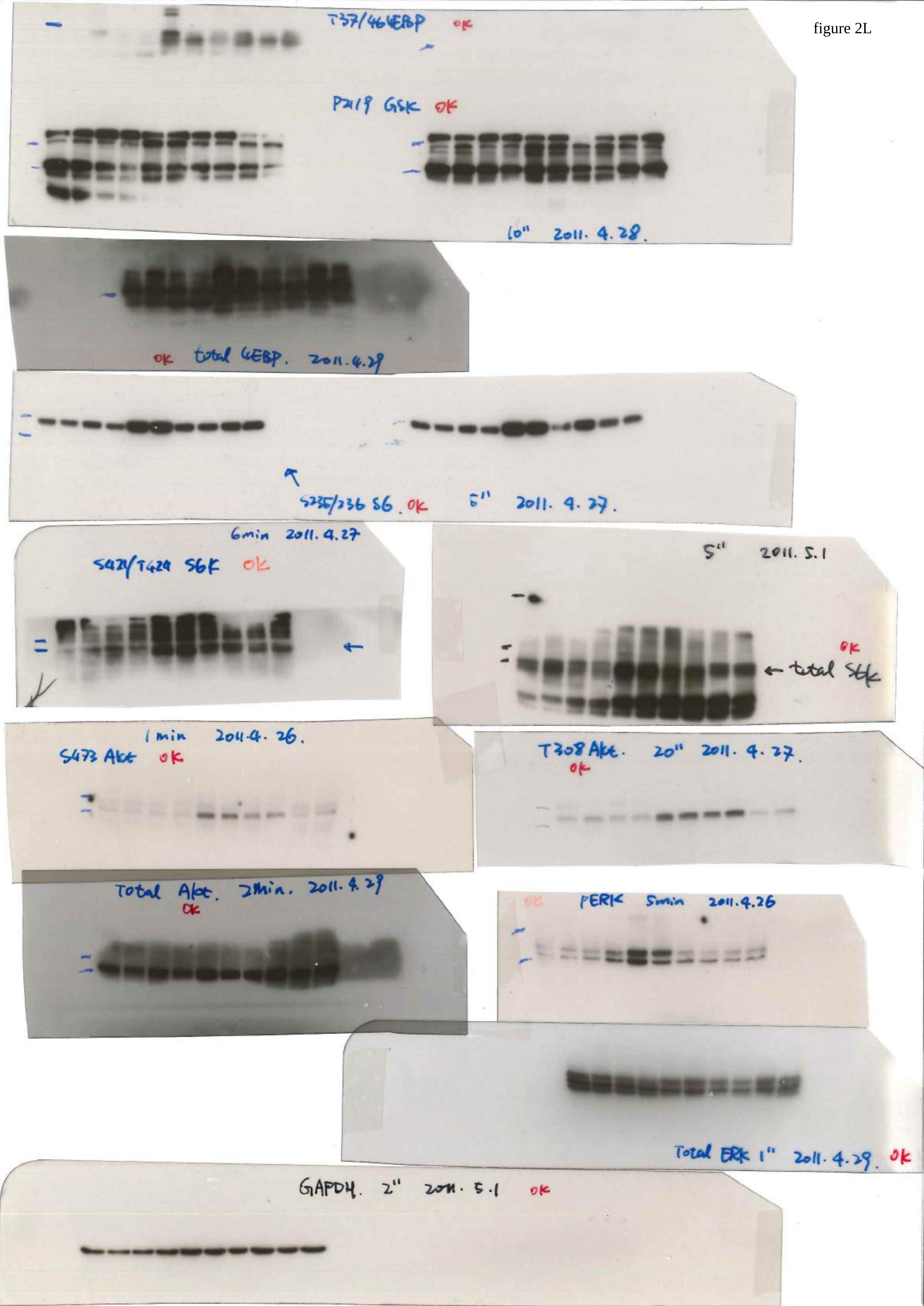
T37/46 4EBP ok
P219 GSK ok
10" 2011. 4. 28.
ok total 4EBP. 2011.4.29
S235/236 S6. ok 5" 2011. 4. 27.
6min 2011.4.27
S421/T424 S6K ok
5" 2011. 5.1
ok
total S6K
1 min 2011.4.26.
S473 Akt ok
T308 Akt. 20" 2011. 4. 27.
ok
Total Akt. 2min. 2011.4.29
ok
ok PERK 5min 2011.4.26
Total ERK 1" 2011. 4.29. ok
GAPDH. 2" 2011. 5.1 ok

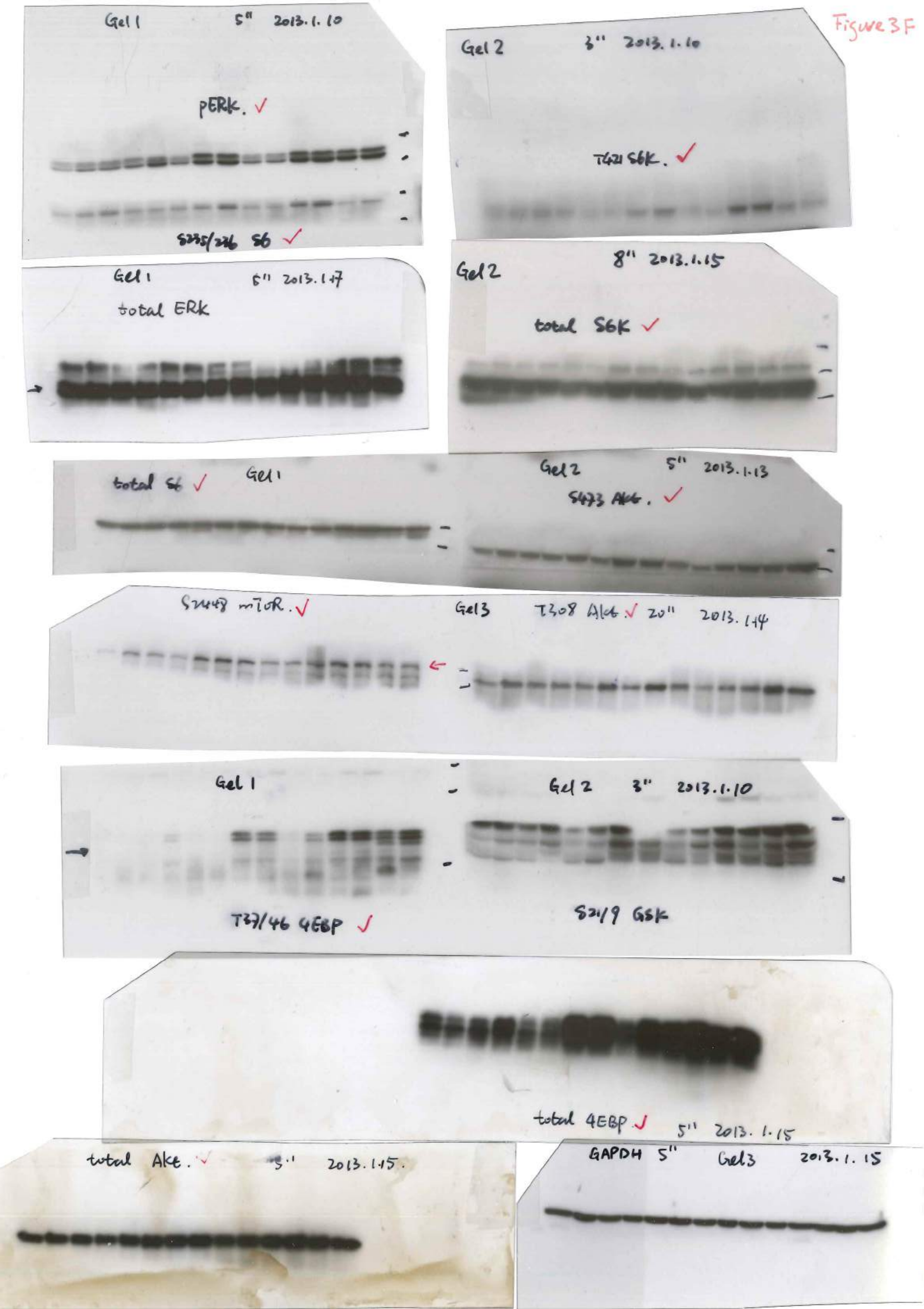
Figure 3F
Gel 1 5" 2013.1.10
pERK. ✓
S235/236 S6 ✓
Gel 2 3" 2013.1.10
T421 S6K. ✓
Gel 1 5" 2013.1.17
total ERK
Gel 2 8" 2013.1.15
total S6K ✓
total S6 ✓ Gel 1
Gel 2 5" 2013.1.13
S473 Akt. ✓
S2448 mTOR. ✓
Gel 3 T308 Akt ✓ 20" 2013.1.14
Gel 1
Gel 2 3" 2013.1.10
T37/46 4EBP ✓
S21/9 GSK
total 4EBP ✓ 5" 2013.1.15
total Akt. ✓ 2013.1.15.
GAPDH 5" Gel 3 2013.1.15

Fig. S1

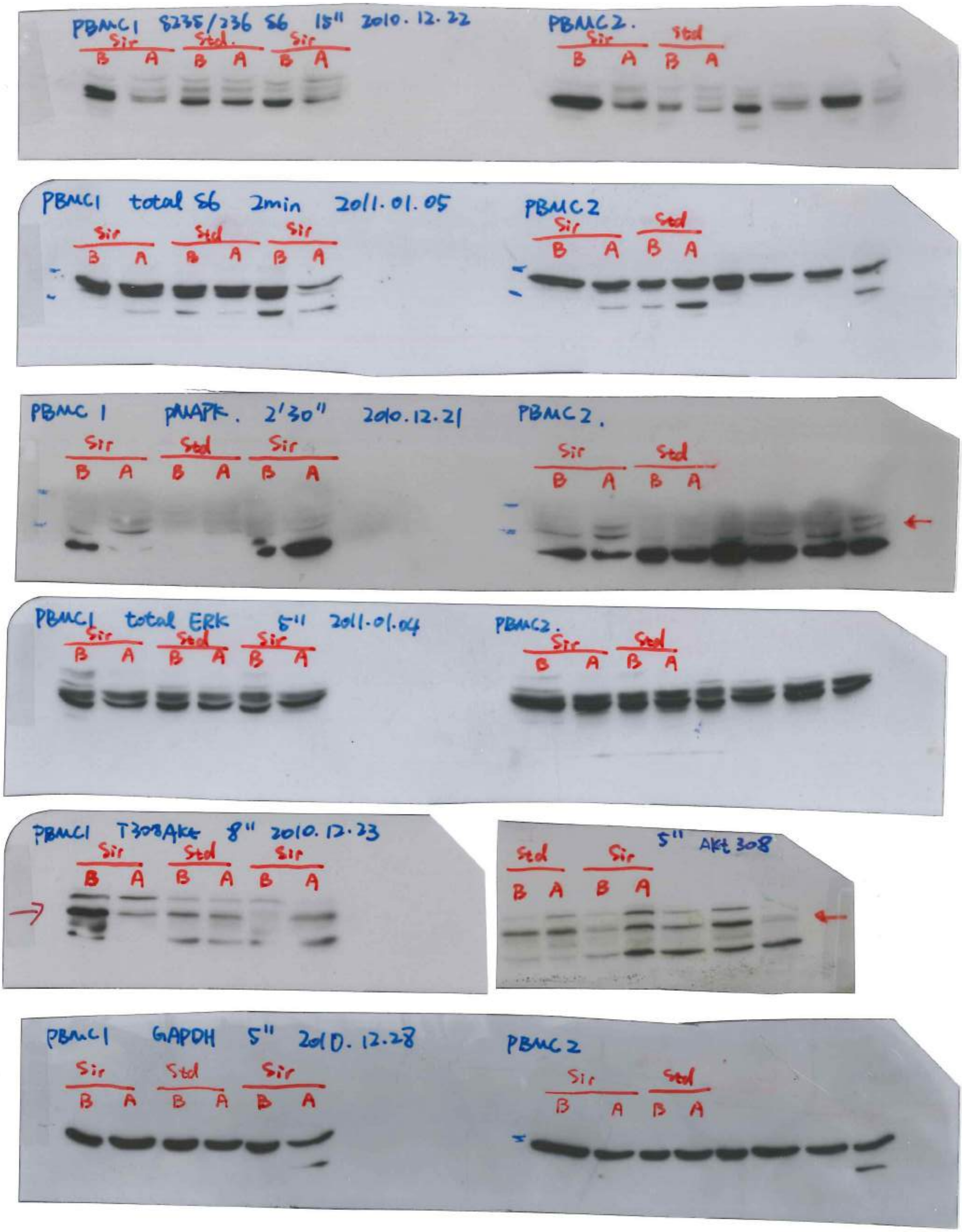
PBMC1 S235/236 S6 15" 2010.12.22
Sir Std. Sir
B A B A B A
PBMC2.
Sir Std
B A B A
PBMC1 total S6 2min 2011.01.05
Sir Std Sir
B A B A B A
PBMC2
Sir Std
B A B A
PBMC 1 pMAPK. 2'30" 2010.12.21
Sir Std Sir
B A B A B A
PBMC2.
Sir Std
B A B A
PBMC1 total ERK 5" 2011.01.04
Sir Std Sir
B A B A B A
PBMC2.
Sir Std
B A B A
PBMC1 T308Akt 8" 2010.12.23
Sir Std Sir
B A B A B A
5" Akt 308
Std Sir
B A B A
PBMC1 GAPDH 5" 2010.12.28
Sir Std Sir
B A B A B A
PBMC2
Sir Std
B A B A

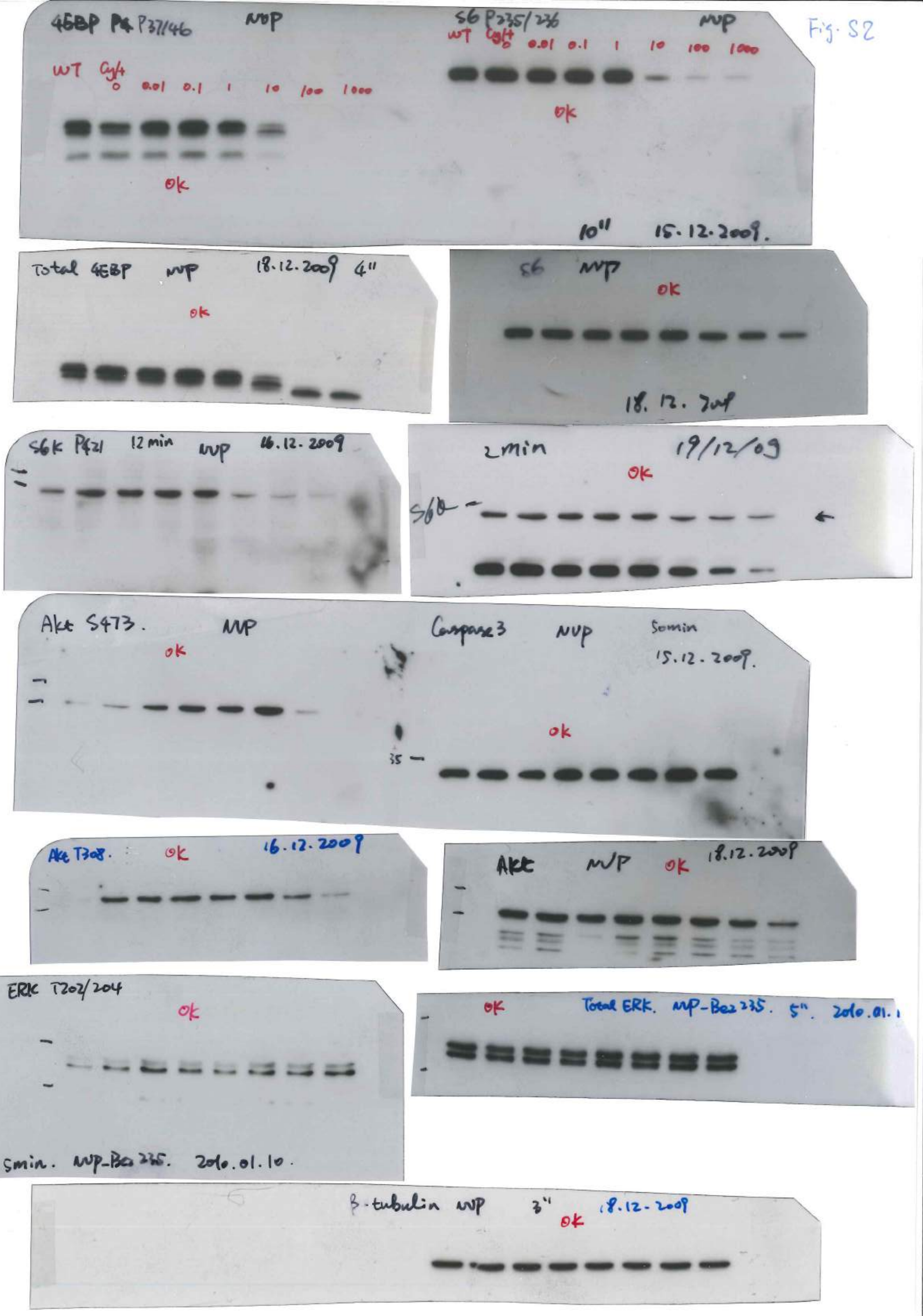
Fig. S2
4EBP P37/46 NVP
WT Cyl4 0 0.01 0.1 1 10 100 1000
ok
S6 P235/236 NVP
WT Cyl4 0 0.01 0.1 1 10 100 1000
ok
10" 15.12.2009.
Total 4EBP NVP 18.12.2009 4"
ok
S6 NVP
ok
18.12.2009
S6K P421 12 min NVP 16.12.2009
2min 19/12/09
OK
Akt S473. NVP
ok
Caspase3 NVP 5omin
15.12.2009.
ok
35
Akt T308. ok 16.12.2009
Akt NVP ok 18.12.2009
ERK T202/204
ok
5min. NVP_Bez235. 2010.01.10.
ok Total ERK. NVP-Bez235. 5". 2010.01.
β-tubulin NVP 3" 18.12.2009
ok

Figure S7

A

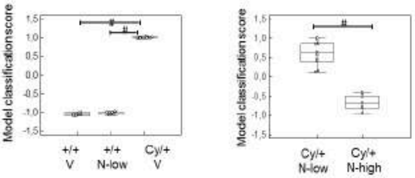
Model classification score
1,5
1,0
0,5
0,0
-0,5
-1,0
-1,5
#
#
+/+
V
+/+
N-low
Cy/+
V
Model classification score
#
Cy/+
N-low
Cy/+
N-high

B

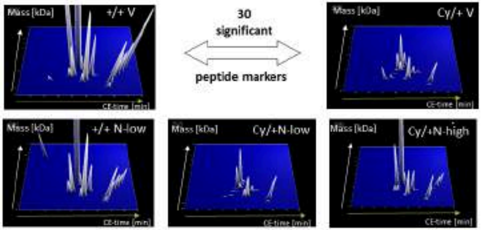
Mass [kDa]
+/+ V
CE-time [min]
30
significant
peptide markers
Cy/+ V
+/+ N-low
Cy/+N-low
Cy/+N-high

SUPPLEMENTARY FIGURE LEGENDS

Figure S1 Blocking the mTOR pathway by sirolimus triggered the activation of ERK and Akt in PBMCs of ADPKD patients. Immunoblots and densitometry readouts of mTOR, PI3K/Akt and PI3K/ERK pathways in PBMCs protein lysates from autosomal dominant polycystic kidney disease (ADPKD) patients allocated to sirolimus (Sir, N=3) and standard care (Placebo, N=2) before (B) and after (A) treatment (interval time = 6 months). * $p < 0.05$, ** $p < 0.01$ (mean ± SD, ANOVA) indicated by brackets.

Figure S2 Effect of NVP-BEZ235 on molecular pathways *in vitro*. Western blot analysis of mTOR pathway, PI3K/Akt and PI3K/ERK pathways treated for 48 h by various concentrations [nM] of NVP-BEZ235 in Cy/+ TEC. Data are representative of three independent experiments.

Figure S3 Quantitative analysis of immunoblots of mTOR, PI3K/Akt and PI3K/ERK pathways *in vivo*. Densitometry readouts of mTOR, PI3K/Akt and PI3K/ERK pathways in kidney tissue lysates from (A) Han:SPRD rats and (B) Pkd1 conditional knock out mice (n= 4 per group). (C) Total body weight of Pkd1 conditional knock out mice. * $p < 0.05$, ** $p < 0.01$, # $p< 0.001$ indicated by brackets (mean ± SD, ANOVA). Data are representative of three independent experiments.

Figure S4 Impact of NVP-BEZ235 treatment on renal morphology and molecular pathways. (**A)** Representative kidney sections of NVP-BEZ235-treated and vehicle-treated rats. Shown are renal histological and immunohistochemical staining with Masson trichrome staining, Ki-67, p-ERK (T202/204) and p-S6 (S240/244) from top to bottom panels, respectively. Quantitative analysis of (**B**) cystic index, (**C**) fibrosis index and (**D**) proliferation index in Han:SPRD rats are displayed. * $p < 0.05$, ** $p < 0.01$, # $p < 0.001$ indicated by brackets (mean ± SD, ANOVA). The number of rat per group is indicated in **Supplementary Tables 1** and **2**

Figure S5 Kidney histology and immunohistochemical staining in Pkd1 conditional knock out mice. Representative kidney sections from NVP-BEZ235-, sirolimus- and vehicle-treated mice. Kidney histology and immunohistochemical staining with PAS, Masson trichrome, Ki-67, p-ERK (T202/204) and p-S6 (S240/244) are displayed from top to bottom panels. Quantitative analysis of (B) cystic index, (C) fibrosis index and (D) proliferation index in Pkd1 conditional knock out mice are displayed. * p < 0.05, ** p < 0.01, # p < 0.001 indicated by brackets (mean ± SD, ANOVA). The number of mice per group is indicated in **Supplementary Tables 4** and **5.**

Figure S6 Original gels from the Western blot experiments. The experiments were performed by cutting the each gel/blot into three pieces: 250-70 kDa, 70-35 kDa and 35-12 kDa for the protein size.

Figure S7 Assessment of the 30-disease related peptide markers in detecting NVP-BEZ235 drug response in PKD rats. (**A**) Box and Whisker plot analysis of peptide- based marker model derived from wild type placebo (+/+ V), wild type treated with NVP 15 mg/kg/day (+/+ N-low) and cystic placebo (Cy/+ V) used as training set after total cross validation with AUC of 1.0, p<0.0001 (**left plot**). Performance of the peptidomic model in the cystic animals treated with lower dosage regime of 15 mg/kg/day (Cy/+ N-low) and higher dosage regime of 50 mg/kg/day (Cy/+ N-high) used as validation set, p<0.0022 (**right plot**).(**B**) Distribution of the 30 peptide markers in each group by specific contour plots. Each consisting of digitally complied (average) data sets in all urine samples from each animal group in 3D depiction. Molecular mass of the polypeptides is presented in logarithmic scale (0.8-20kDa) that is plotted against CE migration time (15-60 min) with MS signal intensity on z-axis.

Supplementary Tables

Table S1 NVP-BEZ235 effect on kidney morphology in male Han:SPRD rats

	+/+ Vehicle n=5	*+/+ NVP-low* n=7	*Cy/+ Vehicle* n=8	*Cy/+ NVP-low* n=8	*Cy/+ NVP-high* n=5
**Cystic index (%) (n)**	3.93 ± 1.85 (5)	3.53 ± 1.52 (6)	32.86 ± 7.1 (7)	20.8 ± 4***(8)	13.18 ± 4.03***(5)
**Ki-67 positive nuclear index (%) (n)**	1.57 ± 0.57 (4)	1.15 ± 0.43 (5)	9.7 ± 4.36 (8)	4.41 ± 0.76**(6)	2.14 ± 0.46***(5)
**Fibrosis index (%) (n)**	8.71 ± 1.51 (3)	8.99 ± 6.29 (5)	24.79 ± 8.15 (8)	17.47 ± 4.95 (8)	12.27 ± 3.69*(5)

* P< 0.05, ** P<0.01, ***P<0.001 vs. Cy/+ Vehicle group

Table S2 NVP-BEZ235 effect on renal function in male Han:SPRD rats

	Time Point (weeks)	*+/+ Vehicle* n=5	*+/+ NVP-low* n=7	*Cy/+ Vehicle* n=8	*Cy/+ NVP-low* n=8	*Cy/+ NVP-high* n=5
**2K/TBW (%)**	9w	0.75 ± 0.08	0.79 ± 0.05	2.23 ± 0.24	1.72 ± 0.18***	1.28 ± 0.26***
**TBW (g)**	4w	90.6 ± 18.74	102.14 ± 22.23	115.5 ± 13.2	116.13 ± 10.88	91 ± 4.24
	6.5w	202 ± 12.96	189.86 ± 15.03	219.5 ± 9.75	193.75 ± 11.74**	146.2 ± 6.42***
	9w	387.56 ± 13.57	257.86 ± 17.28	301.11 ± 10.98	257.31 ±13.72***	223.47 ± 11.01***
**BUN (mg/dL)**	4w	15.58 ± 1.28	15.06 ± 2.46	17.79 ± 4.25	17.3 ± 2.68	15.3 ± 0.88
	6.5w	15.19 ± 1.58	15.73 ± 2.36	25.35 ± 2.58	22.38 ± 3.26*	14.4 ± 1.81***
	9w	17.37 ± 2.02	15.49 ± 1.43	40.58 ± 2.4	31.76 ± 2.39***	15.52 ± 1.8***
**Serum creatinine (mg/dL)**	4w	0.4 ± 0.04	0.39 ±0.01	0.39 ± 0.02	0.38 ± 0.01	0.38 ± 0.01
	6.5w	0.41 ± 0.02	0.4 ± 0.02	0.4 ± 0.02	0.41 ± 0.03	0.42 ± 0.06
	9w	0.44 ± 0.02	0.44 ± 0.02	0.59 ± 0.04	0.54 ± 0.04**	0.44 ± 0.03***
**Urinary albumin/creatinine (mg/mmol)**	9w	1.88 ± 0.83	2.49 ± 1.18	45.22 ± 20.83	14.06 ± 9.78***	2.06 ± 0.69***

2K/TBW% denotes two kidneys weight as a percent of the total body weight.

* P< 0.05, ** P<0.01, ***P<0.001 vs. Cy/+ Vehicle group

Table S3 Characteristics of the 13 sequenced peptide markers included in the SVM-based model

Peptide ID	Mass	Ce-time	Sequence	Protein name	Start	Stop	p-value (adj)	Mean intesity cystic animals	Mean intensity wild type animals	Reg. factor
**10648**	1435,725	39,60707	SpGSPGPDGKTGPpGP	Collagen alpha-1(I) chain	532	547	2,10E-04	1089,24	2692,02	0,40
**19323**	2259,259	37,03559	EPPTGPFVEPPDLFFLKTTK	Proline-rich protein	1304	1323	2,10E-04	1609,77	12763,06	0,13
**19894**	2330,291	37,37787	AEPPTGPFVEPPDLFFLKTTK	Proline-rich protein	2037	2057	2,10E-04	205,17	4383,31	0,05
**9014**	1321,729	30,59061	SGGSQLKSFGQVK	Seminal vesicle secretory protein 2	125	137	2,10E-04	688,04	0,00	688,04
**17050**	2012,961	36,86377	DEQYPDATDEDLTSRMK	Osteopontin	163	179	2,10E-04	433,07	0,00	433,07
**17451**	2054,999	35,61806	GRPGPmGPpGSGGLKGEpGDMG	Collagen alpha-1(V) chain	575	596	2,10E-04	840,98	0,00	840,98
**14518**	1753,766	43,58018	DEQYPDATDEDLTSR	Osteopontin	163	177	3,15E-04	146,58	1,43	102,49
**7299**	1214,582	48,14924	GPpGPpGPpGPPSG	Collagen alpha-1(I) chain	1170	1183	9,60E-04	1064,71	1988,60	0,54
**9411**	1347,674	38,50236	ASDSSINWNNLK	Serotransferrin	455	466	9,60E-04	67,43	457,99	0,15
**9722**	1367,68	48,79485	PpGPpGPpGPpGPPS	Collagen alpha-1(I) chain	1168	1182	9,60E-04	414,44	994,14	0,42
**10709**	1439,72	39,20164	ATGFpGAAGRVGPpGP	Collagen alpha-1(I) chain	862	877	9,60E-04	48,90	632,77	0,08
**14007**	1705,899	41,45552	PpGPAGQpGDKGEGGApGL	Collagen alpha-1(III) chain	768	786	9,60E-04	400,76	1701,49	0,24
**16337**	1936,085	34,91943	TGPFVEPPDLFFLKTTK	Protein Prp2l1	309	328	9,60E-04	97,27	3604,82	0,03

Given are internal peptide ID, frequency and mean amplitude of the cystic placebo and wild type animal models (wild type placebo + wild type treated with 15 mg/kg/day NVP) in training set, adjusted P-value according Benjamini and Hochberg for comparison cystic placebo and wild type animal models, regulation factor (mean amplitude in cystic placebo towards wild type animal models) if applicable amino acid sequence, amino acid start-stop position and precursor protein name.

Table S4 NVP-BEZ235 and everolimus effect on polycystic kidney disease progression in male Han:SPRD rats

	Treatment time (weeks of age)	*2K/TBW*	*TBW*	*BUN (mg/dL)*	*Serum creatinine (mg/dL)*	*Cystic index (%)*
**Cy/+ NVP-high (n)**	4w ~ 9w	1.28 ± 0.26 (5)	223.47 ± 11.01 (5)	15.52 ± 1.80*** (5)	0.44 ± 0.03*(5)	13.18 ± 4.03(5)
**Cy/+ everolimus (n)**	4w ~ 9w	1.15 ± 0.10 (8)	228.25 ± 14.93 (8)	24.20 ± 4.35 (8)	0.53 ± 0.08 (8)	22.33 ± 6.11 (3)

* P< 0.05, ** P<0.01 vs. Cy/+ everolimus group

Table S5 NVP-BEZ235 and sirolimus effect on the development of ADPKD in *Pkd1* conditional knockout mice.

**Gender**	***V-*** **F n= 2 M n= 6**	***N-*** **F n= 3 M n= 5**	***S-*** **F n= 3 M n= 3**	***V+*** **F n= 5 M n= 5**	***N+*** **F n= 4 M n= 3**	***LN+*** **F n= 4 M n= 3**	***S+*** **F n= 2 M n= 3**
**Total body weight (g)**	17.86 ± 1.51	16.55 ± 1.29	15.3 ± 2.6	10.17 ± 2.37	15.66 ± 1.33***	8.59 ± 1.96	9.56 ± 1.29
**2K/TBW%**	1.25 ± 0.1	1.17 ± 0.07	1.25 ± 0.1	8.06 ± 2.74	2.71 ± 0.67***	5.56 ± 1.05***	3.46 ± 1.41*
**BUN (mg/dL)**	21.5 ± 2.62	21.88 ± 3.91	20.8 ± 4.21	276.1 ± 180.97	63.57 ± 31.5***	225.4 ± 106.32	130 ± 124.29*
**Serum Creatinine (mg/dL)**	0.11 ± 0.01	0.13 ± 0.02	0.09 ± 0.01	0.61 ± 0.8	0.2 ± 0.05***	0.51 ± 0.23	0.28 ± 0.24*

2K /TBW% denotes two kidneys weight as a percent of the total body weight.

* P< 0.05, ** P<0.01, ***P<0.001 vs. V+ group

Table S6 NVP-BEZ235 and sirolimus effect on the development of ADPKD in *Pkd1* conditional knockout mice.

	V-	*N-*	*S-*	*V+*	*N+*	*LN+*	*S+*
**Cystic Index (%) (n)**	2.81 ± 0.77 (5)	2.66 ± 0.53 (5)	1.65 ± 0.42 (5)	51.31 ± 9.96 (8)	22.07 ± 8.87***(7)	50.39 ± 6.34 (6)	37.16 ± 5.57* (5)
**Ki-67 positive nuclear index (%) (n)**	1.46 ± 0.82 (5)	1.75 ± 1.01 (5)	3.32 ± 1.25 (5)	7.38 ± 2.94 (5)	2.79 ± 1.79*(7)	7.06 ± 3.54***(6)	5.15 ± 2.82*(5)
**Fibrosis index (%) (n)**	0.13 ± 0.06 (5)	0.41 ± 0.58 (5)	0.22 ± 0.04 (5)	11.11 ± 6.52 (5)	1.45 ± 0.83***(6)	4.69 ± 1.07**(6)	3.14 ± 0.87***(5)

* P< 0.05, ** P<0.01, ***P<0.001 vs. V+ group

Table S7 Plasma concentration of NVP-BEZ235 in male Han:SPRD rats

	*Day 25** (µmol/l)*	*Day 28*** (µmol/l)*
**+/+ (n=3)**	2.70 ± 1.48	3.17 ± 1.67
**Cy/+ (n=3)**	3.12 ± 0.13	2.76 ± 1.87

* Plasma obtained from NVP-BEZ235 naïve rats, aged 25 days, 1h after 50 mg/kg NVP-BEZ235 was applied by gavage.
** Plasma obtained from rats, aged 28 days, treated for 4 days with 50 mg/kg/day NVP-BEZ235 by gavage,1h after 50 mg/kg NVP-BEZ235 was applied by gavage.
